# Immediate Loading of Implants Placed Immediately in Fresh Sockets: A 10-Year Single-Arm Prospective Case Series Follow-Up

**DOI:** 10.3390/jcm14248830

**Published:** 2025-12-13

**Authors:** Eugenio Velasco-Ortega, Ivan Ortiz-Garcia, Loreto Monsalve-Guil, José López-López, Enrique Núñez-Márquez, Nuno Matos-Garrido, José Luis Rondón-Romero, Álvaro Jiménez-Guerra, Jesús Moreno-Muñoz

**Affiliations:** 1 Comprehensive Dentistry for Adults and Gerodontology, Faculty of Dentistry, University of Seville, 41004 Seville, Spain; evelasco@us.es (E.V.-O.); ivanortizgarcia1000@hotmail.com (I.O.-G.); lomonsalve@hotmail.es (L.M.-G.); nunogarrido@orallagos.pt (N.M.-G.); jolurr001@hotmail.com (J.L.R.-R.); alopajanosas@hotmail.com (Á.J.-G.); je5us@hotmail.com (J.M.-M.); 2 Service of the Medical-Surgical Area of Dentistry Hospital, Faculty of Dentistry, University of Barcelona, 08007 Barcelona, Spain

**Keywords:** immediate placed implants, fresh sockets, immediate loading

## Abstract

**Background.** Implant dentistry is an important treatment option for patients requiring prosthetic rehabilitation after tooth loss. This study reports the evaluation of immediately loaded, immediately placed implants in fresh extraction sockets. **Methods**. Fifty-two partially edentulous patients (27 females and 25 males with mean age of 53.6 years), were treated with 112 Galimplant^®^ implants placed immediately into fresh sockets for prosthodontic rehabilitation. All implants were loaded immediately. Clinical and radiographic parameters related to both the implants and the prosthodontic restorations were followed for 10 years. **Results**. Nine patients (17.3%) had a history of periodontitis, 26.9% were smokers, and 21.1% presented with chronic systemic conditions. The outcomes demonstrated an implant survival and success rate of 97.1%, indicating that immediately placed implants with immediate loading can achieve and maintain successful osseointegration. Three implants were lost during the healing period. The mean marginal bone loss was 1.09 ± 0.75 mm. Mucositis affected 21.4% of implants, and peri-implantitis was observed in 11.6% of implants. Fourteen implants (7.1%) were associated with technical complications, including screw loosening and ceramic chipping. **Conclusions**. The clinical findings of this study indicate favorable long-term outcomes for immediately loaded implants placed in fresh extraction sockets. Both implants and prosthetic restorations demonstrated a success rate of over 92.9% during the observation period.

## 1. Introduction

Dental implants have revolutionized modern dentistry, and implant therapy has become a widely accepted treatment modality for the replacement of missing teeth. Implant-supported prostheses are commonly used in the rehabilitation of partially and completely edentulous patients, achieving significant functional and esthetic success [1,2]. The outcomes of dental implant therapy are closely linked to the process of osseointegration, a sequence of biological events that occurs after the surgical placement of an implant into the alveolar bone. Moreover, the functional loading of implants represents the biomechanical culmination of treatment [3,4].

Long-term studies have demonstrated favorable survival and success rates for implants in the general population, with increased survival reported across a variety of clinical situations and patient groups [5,6]. Both studies present data with more than 10 years of follow-up, with a survival rate of 96.4% at 15 years in the work of Velasco-Ortega et al. (2022) [6] and 91.8% at 16.9 years and 80% at 19.6 years in that of McGlumphy et al. (2019) [5]. Advancements in implant design and biomaterials have expanded clinical indications, including immediate implant placement in fresh extraction sockets and immediate loading protocols [7,8].

Following tooth extraction, the alveolar socket undergoes significant dimensional changes as part of the physiological healing process, with substantial reductions in ridge width and height typically occurring within the first year. Immediate implant placement involves inserting the implant directly into the extraction socket at the time of tooth removal. Immediate placement and restoration require comprehensive diagnostic evaluation and meticulous treatment planning to achieve predictable outcomes. This approach offers several advantages, including reduced treatment time, fewer surgical interventions, and preservation of alveolar bone, all of which may contribute to improved overall results [9,10].

A key determinant of successful immediate implant placement is achieving adequate primary stability. Securing primary stability in fresh extraction sites can be challenging due to the socket morphology. Once an implant is inserted into a fresh socket, its stability depends primarily on the mechanical engagement of the apical bone, as no biological interface initially exists between the implant surface and the surrounding tissues. Subsequent osseointegration develops through bone remodeling and new bone formation, ultimately establishing biological stability [11,12].

In cases involving immediate loading, primary stability becomes even more critical, especially when implants are placed into fresh post-extraction sockets [13]. Immediate implant placement combined with immediate loading is a technically demanding procedure that should be undertaken only by clinicians with advanced training and experience. Its success relies on specialized expertise and careful control of occlusal forces during the healing phase [14]. The most critical requirement is adequate residual bone volume and quality. To minimize complications associated with tooth extraction, implants should be placed immediately following atraumatic extraction. Additionally, clinicians must evaluate the preservation of bone architecture and soft-tissue contours and ensure appropriate immediate prosthetic restoration [15,16,17].

## 2. Materials and Methods

This prospective study included patients treated at the Master’s Program in Implant Dentistry Clinic of the Faculty of Dentistry at the University of Seville, Spain, who required tooth extraction and subsequent replacement with implant-supported prostheses between January 2013 and July 2025.

The study was conducted in accordance with the principles of the Declaration of Helsinki for research involving human participants. Ethical approval was obtained from the University of Seville Ethics Committee, and all participants provided written informed consent before undergoing immediate implant placement.

The inclusion criteria required systemically healthy patients with good oral hygiene. Exclusion criteria included uncontrolled chronic systemic disease, smoking ≥10 cigarettes per day, bruxism, active periodontal disease, and alcohol or drug abuse.

All implant sites presented with ≤4 mm of bone beyond the root apex to ensure adequate primary stability. Additional requirements included atraumatic tooth extraction, preservation of the buccal plate, and an insertion torque of ≥35 N·cm. Treatment planning incorporated diagnostic casts to evaluate intermaxillary relationships, periapical and panoramic radiographs, and clinical photographs. Patients were informed of all available implant-based treatment options for tooth replacement and provided consent for immediate implant placement with immediate loading.

One hour before surgery, patients received prophylactic antibiotic therapy consisting of 500 mg of amoxicillin and 125 mg of clavulanic acid. This regimen was continued postoperatively at a dosage of three capsules per day for five days. Following the procedure, patients were instructed to use a chlorhexidine mouthwash twice daily for 15 days. Ibuprofen (600 mg, twice daily) was prescribed as needed for pain or inflammation. All surgical procedures were performed under local anesthesia using 4% articaine with 1:100,000 epinephrine.

A flapless approach was used in all cases, and extractions were carried out with elevators to minimize surgical trauma. Particular attention was given to preserving the integrity of the buccal bone wall. After extraction, each socket was thoroughly curetted, and the implant bed was prepared according to the established protocol. None of the sites exhibited periapical pathology. Implant site preparation was performed with standard drills, maintaining alignment with the palatal bony wall, and all implants were positioned at least 4 mm beyond the root apex. The coronal margin of the implant was placed at the level of the buccal bone crest. IPX^®^ screw-type implants (Galimplant^®^, Sarria, Spain) featuring a sandblasted, acid-etched surface and internal connection were used for all placements. No grafting materials or barrier membranes were used.

Following surgery, all patients immediately received abutments and provisional prosthetic restorations. For single-tooth replacements, acrylic resin–cemented crowns were used. Immediate loading was performed only when an insertion torque of ≥35 N·cm was achieved. The provisional crowns were removed three months after implant placement. Impressions were then taken using silicone materials with open customized trays. Definitive metal–ceramic restorations were subsequently cemented onto the osseointegrated implants.

**Figure 1:**
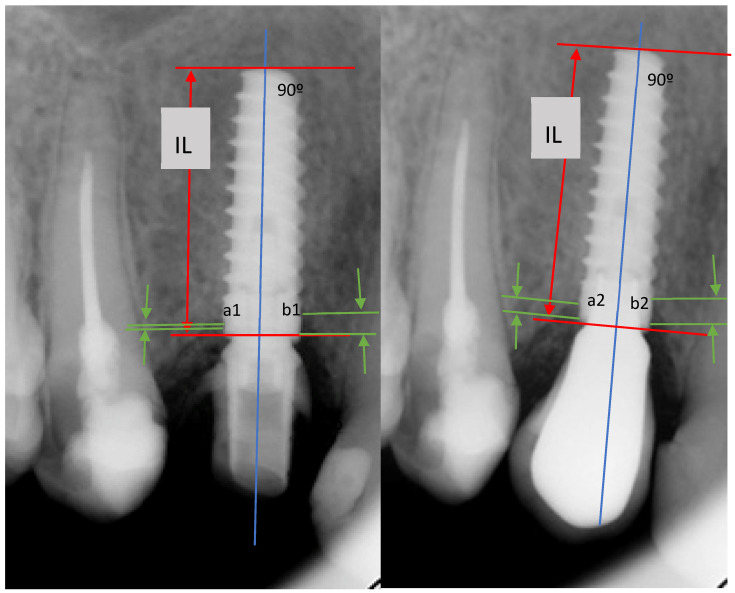
Diagram illustrating the method used to calculate mesial and distal marginal bone loss. IL: implant length. The blue line is drawn perpendicular to the axis of the implant and at the midpoint (mesial-distal) of the implant. The red arrow indicates the known implant length, while the green arrows reference the crestal bone and marginal bone loss, enabling comparison at different times in this study. a1: distal bone level in evaluation 1; a2: mesial bone level in evaluation 2; b1: distal bone level in evaluation 1; b2: mesial bone level in evaluation 2.

Implant survival was assessed based on the presence of stability and the absence of peri-implant radiolucency, mucosal suppuration, or pain. Follow-up evaluations were performed three months after implant placement and annually thereafter. During these visits, the implants and prostheses were professionally cleaned and examined both clinically and radiographically. Marginal bone loss was evaluated using digital periapical radiographs obtained perpendicular to the long axis of the implant. Changes in bone level were determined by comparing the radiograph taken at the 1-year follow-up with those obtained at each subsequent annual visit. To assess marginal bone loss, both mesial and distal, we used the diagram shown in Figure 1, and which we described in previous clinical study [6]. The following patient- and implant-related variables were recorded: age, sex, smoking habits (<10 cigarettes/day), history of periodontitis, reason for tooth extraction, implant site, implant diameter and length, and any biological or technical complications. Patient-related variables comprised the first four parameters, whereas implant-related variables comprised the remaining four.

All available examination data were analyzed using the SPSS software package (SPSS 11.5.0, SPSS Inc., Chicago, USA (Illinois)). Descriptive statistics (frequencies, mean, standard deviation, and sum) were performed on the clinical findings of the study, with reference to the patients’ demographic variables (age and sex), oral variables (e.g., periodontal history), systemic variables (e.g., smoking habits, medical history), surgical and implant characteristics (diameter, length, complications, and losses), as well as the functional load with the prosthodontic restorations and the clinical follow-up of the implants. Contingency tables were created for all qualitative variables, which were analyzed using the chi-square test. Frequency and percentage were calculated for each cell and column. Quantitative variables were analyzed using the variance test when the distribution was normal, in relation to all qualitative variables. A non-parametric test was performed on numerical variables with a non-normal distribution in relation to all qualitative variables. The Mann–Whitney U test was used for dichotomous variables and the Kruskal–Wallis test for variables with more than two categories. A significant level of 5% was adopted for all statistical analyses.

## 3. Results

The study population consisted of 52 consecutively treated patients, 27 females and 25 males, ranging in age from 40 to 69 years (mean age, 53.6 years). No statistically significant differences were observed with respect to sex or age (chi-square test, *p* = 0.83408). No intraoperative problems were recorded, the existing gap was not filled with biomaterial, and all provisional crowns were left in infraocclusion until the final crowns were inserted. Nine patients (17.3%) had a history of periodontitis. Fourteen patients (26.9%) were smokers (Table 1). Eleven patients (21.1%) exhibited chronic systemic conditions, including hypertension, diabetes, and cardiovascular diseases.

A total of 112 implants were placed immediately following tooth extraction. The indications for extraction included caries with endodontic treatment failure, periodontal disease, and tooth fracture. Implant sites consisted of incisors (60 maxillary and 12 mandibular) and premolars (24 maxillary and 16 mandibular). The mean insertion torque was 47 N·cm ± 12 N·cm, with no difference by sex or age and with a mean torque of 45.2 N·cm in the maxilla and 48.1 in the mandible.

Sixteen implants (14.3%) measured 10 mm in length, 69 implants (61.6%) measured 12 mm, and 27 implants (24.1%) measured 14 mm. A total of 28 implants (25.0%) had a diameter of 3.5 mm, 80 implants (71.4%) had a diameter of 4.0 mm, and 4 implants (3.6%) had a diameter of 5.0 mm. In total, 3 implants (2.9%) failed during the initial healing phase and were classified as early failures (Table 2 and Table 3). All patients who experienced implant loss were successfully retreated with new implants. The cumulative survival rate for all implants was 97.1%.

The mean follow-up period was 127.7 ± 44.1 months (range: 60–166 months). The mean marginal bone loss (MBL) was 1.09 ± 0.75 mm, ranging from 0 to 2.6 mm between implant placement and the 10-year follow-up evaluation (Table 4). MBL was associated with several clinical variables. Smokers exhibited significantly greater marginal bone loss (1.11 ± 0.90 mm) compared with nonsmokers (0.93 ± 0.84 mm) (Mann–Whitney U test; *p* = 0.0217). Maxillary implants also showed significantly greater MBL (1.05 ± 0.75 mm) than mandibular implants (0.92 ± 0.79 mm) (Mann–Whitney U test; *p* = 0.0437).

During the follow-up period, 24 implants (21.4%) were associated with peri-implant mucositis. Mucositis occurred significantly more frequently in patients with a history of periodontitis (chi-square test, *p* = 0.00002) and in smokers (chi-square test, *p* = 0.02670). Thirteen implants (11.6%) developed peri-implantitis (Table 5). Peri-implantitis was significantly more common among smokers (chi-square test, *p* = 0.00010) and in patients with chronic systemic diseases (chi-square test, *p* = 0.00837).

The mean peri-implant probing depth was 2.8 mm ± 0.6 mm. The distribution of the mean peri-implant probing depth showed significant normality of the numerical variables according to the Kolmogorov–Smirnov test (*p* = 0.0044). The only variables that showed significant differences were: The mean peri-implant probing depth was 3.3 mm ± 3.8 mm in patients with periodontitis and 2.5 mm ± 0.4 mm in patients without periodontitis. These differences were significant according to the analysis of variance (*p* = 0.0000). Among smokers, the mean probing depth was 3.4 mm ± 0.4 mm, and 2.1 mm ± 0.7 mm among non-smokers (*p* = 0.0000). 13 patients presented with bleeding on probing in at least one implant (25%), and 25 of the 112 implants placed showed bleeding on probing (22.3%). Regarding the patients’ periodontal history, bleeding was present in all 9 patients with periodontitis (69.2%), while 4 patients did not have periodontal disease (30.7%). These differences were significant according to the chi-square test (*p* = 0.01916). Regarding smoking status, 5 patients were smokers (35.7%) and 9 were non-smokers (64.3%). These differences were also significant according to the chi-square test (*p* = 0.02923). Nine patients (13%) presented with suppuration on probing of their implants. Regarding the patients’ periodontal history, 8 patients had periodontal disease (88.9%) and one patient did not (11.1%). There were significant differences according to the chi-square test (*p* = 0.01070). Focusing on the smoking patients, all 9 patients with suppuration were smokers with a history of periodontal disease (Table 6).

The provisional crowns were removed three months after implant placement. Definitive prosthetic rehabilitation consisted of single metal–ceramic cemented crowns. These restorations remained functional throughout the 10-year follow-up period, with a cumulative success rate of 92.9%. Fourteen implants (7.1%) were associated with technical complications, including screw loosening and ceramic chipping (Table 4).

## 4. Discussion

The immediate placement and restoration of dental implants in fresh extraction sockets require thorough diagnosis and meticulous treatment planning to achieve optimal functional and esthetic outcomes. For patients requiring tooth extraction, immediate implant placement combined with a provisional restoration can offer a relatively rapid treatment course and favorable esthetics. However, this technique is complex and requires specific clinical conditions to ensure predictable success. Among these, adequate residual bone volume is a critical prerequisite. This approach offers several advantages, including reduced overall treatment time and decreased surgical trauma, leading to increased patient satisfaction. Nevertheless, clinicians should limit the use of this technique to cases in which the anatomical and clinical conditions are clearly favorable [7,8,9,10,11,12].

In this study, a total of 112 immediate implants were inserted in fresh extraction sockets for immediate loading, resulting in a cumulative survival rate of 97.1%. All cases involved tooth extraction followed by flapless immediate implant placement and immediate loading. The clinical outcomes observed over the 10-year follow-up period demonstrate that implants placed immediately after extraction and restored with immediate loading achieve favorable long-term results and maintain stable peri-implant conditions [14]. These findings are consistent with several studies reporting that immediate post-extraction implant placement combined with immediate loading of provisional restorations is a reliable protocol associated with high success rates [14,15,17,18].

A recent study evaluated clinical outcomes in 20 patients who received immediately placed tapered implants with immediate loading using patient-specific abutments for single-tooth replacement in the anterior maxilla and also assessed patient-reported outcomes. Implant survival at both 24 and 36 months was 100%, with no technical or mechanical complications reported. Patients expressed a high level of overall satisfaction at the 36-month follow-up [17]. Another clinical study assessed outcomes in 30 patients treated with 43 implants placed immediately into fresh and infected sites and immediately loaded. All implants were inserted with a torque ≥35 N·cm. The mean follow-up period was 6 years (range: 1–8 years), and 65% of implants were followed for more than 5 years. No implant failures occurred, and the implant success rate was 93% [18]. In this study, with an average insertion torque of 47 N·cm (and always ≥35 N·cm), we obtained a success rate of 97.1, unrelated to the insertion torque. Chakraborty et al. (2025) [19] analyzed insertion torques based on bone quality and with figures between 26.4 and 48.2 N·cm, obtains no differences in terms of success.

The placement of immediate implants in fresh extraction sockets, compared with placement in healed sites, remains a topic of considerable interest and ongoing debate in implant dentistry. Variability in results often stems from differences in study designs and methodologies across clinical trials and systematic reviews [9,11,15]. Despite these discrepancies, the immediate post-extraction implant technique has become increasingly common in daily clinical practice [20]. Several review studies suggest that implants placed in fresh extraction sockets may present a significantly higher risk of failure and complications compared with implants placed in healed bone [8,9,21,22]. One possible explanation is the discrepancy between the shape and dimensions of the extraction socket and the macrogeometry of the implant. This mismatch can compromise primary stability, as the implant typically engages the bone only at the apical region rather than along the full length of the socket walls. For this reason, it is recommended that implants be inserted at least 3–5 mm beyond the apical bone to achieve adequate anchorage. Additional challenges arising from the lack of adaptation between the implant and the socket walls include the presence of bone defects, which may necessitate simultaneous alveolar preservation procedures with bone graft substitutes [21,22].

However, several systematic reviews on immediate implant placement have reported high survival rates and clinical outcomes comparable to those of implants placed in healed sites [23,24,25]. These findings are supported by a large retrospective study that analyzed patient data from 10 university dental clinics over a period of more than 10 years, evaluating dental implant treatment outcomes [10]. In total, records from 20,842 patients and 50,333 implants were reviewed. Both immediate and delayed implant placement were shown to be viable therapeutic approaches with predictable results. The survival rate of implants placed immediately after extraction (98.4%) was comparable to that of implants placed in healed bone (98.6%) [10]. In the present study, all immediately placed implants were restored with provisional crowns that were removed three months after insertion, followed by definitive metal–ceramic prosthetic restorations. These restorations remained functional throughout the 10-year follow-up period, resulting in a cumulative success rate of 92.9%. A total of 64.3% of the implants were placed in anterior sites (incisors), while 35.7% were placed in posterior sites (premolars).

Previously, immediate implant placement and immediate loading were largely limited to the esthetic zone, particularly the anterior maxilla [7,8,15]. However, in recent years, interest has grown in applying immediate placement protocols in the posterior region, with the objective of minimizing bone remodeling and reducing the need for bone augmentation procedures [14]. A crucial factor for the success of immediate implant placement is achieving adequate primary stability. More recently, the placement of implants in fresh extraction sockets of maxillary and mandibular molars has also been incorporated into clinical practice [13,26]. In our study, 42 implants (37.5%) were placed in the premolar area (24 in the maxilla and 16 in the mandible) with no significant difference in terms of bone loss and/or bleeding on probing.

Immediate implants may offer several advantages, including improved preservation of peri-implant tissues, which may help reduce subsequent bone resorption. This concept is particularly important in anterior regions, where maintaining the soft- and hard-tissue architecture contributes to a more favorable emergence profile and superior esthetic outcomes. In some cases, provisional crowns placed immediately can even seal fresh extraction sockets, supporting the preservation of tissue contours from the earliest stages of healing [27,28].

To preserve the bone architecture and soft-tissue profile in the maxillary esthetic zone, immediate prosthetic restoration on immediately placed implants following atraumatic extraction is often the preferred approach [16,29]. Abutment placement is a critical step in the immediate loading of post-extraction implants that achieve adequate primary stability. An insertion torque of at least 35 N·cm is generally recommended. The design of the definitive restorations should follow both functional and esthetic principles, ensuring that the prosthesis can withstand occlusal forces while harmonizing with the patient’s smile [14,15,16]. In this regard, although we have not done a detailed analysis of the data, all patients were satisfied with the aesthetic results obtained.

The importance of immediate restorations on immediately placed implants has been highlighted by several studies demonstrating their beneficial influence on peri-implant tissues [30,31,32]. A prospective study reported that the use of a prosthetic template can effectively assist in the design of provisional crowns for immediately placed implants, ensuring proper maintenance of contact areas with adjacent teeth [30]. A clinical trial evaluated 55 patients treated with 60 implants placed in extraction sockets and restored with immediate provisionalization. Definitive prostheses were delivered within the first year. The cumulative survival rate at 2 years was 98.3%, with only one implant failing within the first 3 months. Patient-reported outcomes—including function, esthetics, and self-esteem—showed significant improvement following treatment [31]. Another prospective study assessed the esthetic outcomes of 64 anterior maxillary single-tooth implants placed according to immediate post-extraction and immediate loading protocols. After 3 years of function, the implant success rate was 100%, with no implant failures reported. These findings, which align with our result, support the conclusion that immediate loading of post-extraction implants is a predictable technique for achieving favorable functional and esthetic results [32].

MBL is considered a key clinical parameter for the long-term success of immediately placed implants [12,29,31,33]. Multiple factors have been associated with MBL. Patient-related variables (such as a history of periodontitis, smoking, and diabetes), implant-related variables (including surgical technique, implant position, macrogeometry, and surface characteristics), and prosthetic variables (such as timing of loading, abutment selection, and type of prosthetic rehabilitation) may all contribute as risk factors for increased MBL [34,35,36].

The present study reports a mean MBL of 1.09 ± 0.75 mm over a 10-year follow-up period. MBL was significantly greater in smokers and in maxillary implants. These findings are consistent with a recent study that radiographically measured marginal bone loss of 0.93 ± 0.83 mm at one year and 1.04 ± 0.97 mm at three years after implant placement. That study also demonstrated a clear trend of higher MBL in implants placed in the upper jaw and in smokers, with the combination of these two factors further increasing the likelihood of early marginal bone remodeling [37].

Moreover, the design and size of the abutment may play an important role in MBL associated with immediately placed implants. A two-year retrospective analysis was conducted on patients with single-tooth edentulous sites in the premolar, cuspid, and incisor regions who were treated with fresh-socket implants and immediate preformed anatomical healing caps. The design of the healing abutment was found to be crucial for preserving the emergence profile immediately after extraction and implant placement. The length of the implant collar used in conjunction with the immediate healing abutment appeared to influence the preservation of the alveolar crest, contributing to predictable final outcomes [35].

These factors associated with marginal bone loss (MBL) are further supported by findings from a study that evaluated MBL in immediately placed dental implants over a follow-up period of up to 36 months. In that study, smokers exhibited greater MBL as early as 2 months post-placement. Implants placed in the maxilla showed significantly higher MBL at both 12 and 24 months. Regarding abutment type, anti-rotational abutments used for single-unit prostheses demonstrated significantly greater MBL at 12 months compared with rotational abutments used for multiple-unit restorations. Differences related to abutment height were also observed at 6 and 12 months [34].

In the present study, 9 patients (17.3%) had a history of periodontitis. Implants placed in these patients exhibited greater MBL. These findings are consistent with those of a one-year retrospective study that included 95 patients and 234 implants treated with immediate implant placement [38]. Clinical assessments in that study demonstrated a significant increase in MBL with greater periodontal disease severity. MBL was significantly higher in patients with Stage IV periodontitis compared to patients with Stages II and III, indicating that both the severity and progression of periodontal disease may influence clinical outcomes in immediately placed implants in periodontitis patients [38].

Mucositis and peri-implantitis are the most common biological complications affecting the soft and hard tissues supporting dental implants. Peri-implantitis is characterized by inflammation of the peri-implant tissues accompanied by progressive loss of supporting bone [39,40]. In the present study, mucositis occurred significantly more frequently in patients with a history of periodontitis and in smokers, affecting 24 implants (21.4%). Thirteen implants (11.6%) developed peri-implantitis. Peri-implantitis was significantly more common in smokers and in patients with chronic systemic diseases. A prospective observational study evaluated the clinical success of immediately placed implants used for single-tooth replacement over a 3-year period. Although most patients maintained good oral hygiene, the incidence of peri-implant mucositis was reported at 31.3% at 24 months and 25.0% at 36 months [17]. Our data on peri-implantitis and mucositis, while consistent with other published long-term follow-up studies [41], are high for a controlled population. In our case, it is worth noting that 45 of the 52 patients (86.5%) attended their annual visits, and only 2 had continuous follow-up for 3 years. The three failed implants occurred in these two patients.

A prospective multicenter cohort study evaluated single-tooth immediate implant placement and loading in esthetic areas [42]. Data were collected from 215 implants placed in 215 patients across 15 centers over 2 years. Although peri-implant tissues remained stable after final restoration and patient satisfaction was high, 5 cases of mucositis (2.3%) and 1 case of peri-implantitis (0.5%) were observed during the follow-up period [42]. However, it is worth noting in this study that the follow-up is only for two years and that the average number of controls per center is only 14.3 patients.

Fourteen implants (7.1%) in the present study were associated with technical complications, including screw loosening, ceramic chipping, and prosthetic screw fracture. Technical complications are commonly reported in studies involving restorations supported by immediately placed implants in fresh extraction sockets [12,18]. One clinical study reported the outcomes of immediate implant placement in fresh sockets followed by provisional restoration delivered 24 h after surgery [18]. The mean follow-up period was 6 years. In that study, no single-unit provisional restorations were placed in occlusion; only multi-unit provisional prostheses were used. The definitive prosthesis was fabricated after peri-implant tissue maturation guided by the provisional restoration. The prosthetic survival rate was 93%, and the rate of technical complications (7%) was primarily due to veneer material fracture [18]. Another study evaluated 10 patients treated with immediately placed implants restored with screw-retained provisional restorations delivered on the same day the implants were uncovered [12]. Definitive crowns were fabricated after 3 months. Over a 10-year period, 2 patients (20%) experienced technical complications: one required a new restoration due to a fracture, and one experienced debonding of the abutment restoration [12].

## 5. Conclusions

This 10-year follow-up clinical study demonstrated that immediate loading of immediately placed implants in fresh extraction sockets, including in medically controlled patients, is a clinically predictable treatment option when strict patient selection criteria and carefully planned clinical procedures are applied. The implants and the prostheses showed success rates of 97.1% and 92.9%, respectively. Peri-implant bone tissue response was favorable, with a mean marginal bone loss of 1.09 ± 0.75 mm and mean peri-implant probing depth of 2.8 mm ± 0.6 mm.

## Figures and Tables

**Table 1:** Description of patients’ characteristics.

	n	%
Females	27	51.9
Males	25	48.1
History of periodontitis	9	17.3
Smokers	14	26.9
Medical conditions	11	21.1

n = patient.

**Table 2:** Description of implants’ characteristics. N = 112.

	n	%
10 mm implant length	16	14.3
12 mm implant length	69	61.6
14 mm implant length	27	24.1
3.5 mm implant diameter	28	25
4 mm implant diameter	80	71.4
5 mm implant diameter	4	3.6
Loss of implant	3	2.9

n = implant.

**Table 3:** Number of implants implanted by length and diameter.

Implant Length (mm)	10			12			14			Total
Implant diameter (mm)	3.5	4	5	3.5	4	5	3.5	4	5	
Maxillary premolar	0	1	2	7	8	0	1	5	0	24
Mandibular premolar	0	1	1	4	8	0	0	2	0	16
Maxillary incisor	10	0	0	3	29	0	2	16	0	60
Mandibular incisor	0	0	1	0	10	0	1	0	0	12
Total	10	2	4	14	55	0	4	23	0	112
	16			69			27			

**Table 4:** Description of marginal bone loss.

	+	−
History of periodontitis	1.07 ± 0.87 mm	0.95 ± 0.66 mm
Smoking	1.11 ± 0.90 mm	0.93 ± 0.84 mm
Systemic diseases	1.02 ± 0.89 mm	0.89 ± 0.78 mm

**Table 5:** Description of patients with complications.

	n	%
Mucositis	24	21.4
Peri-implantitis	13	11.6
Technical complications	8	7.1
Implant loss	3	2.9

n = implant.

**Table 6:** Description of Mid-peri-implant probing, Bleeding on probing, Suppuration on probing in patients with periodontal history and/or smokers.

	History of Periodontitis	No History of Periodontitis	*p* Value
Mid-peri-implant probing	3.3 ± 3.8 mm	2.5 ± 0.4 mm	0.0000
Bleeding on probing	9 patients	4 patients	0.0191
Suppuration on probing	8 patients	1 patient	0.0140
	Smoking	No Smoking	
Mid-peri-implant probing	3.4 ± 2.6 mm	2.6 ± 0.7 mm	0.0000
Bleeding on probing	5	9	0.0292
Suppuration on probing	9	0	0.0000

## Data Availability

There are no additional data available, please contact the authors if needed.
